# Consensus dynamics in online collaboration systems

**DOI:** 10.1186/s40649-018-0050-1

**Published:** 2018-02-01

**Authors:** Ilire Hasani-Mavriqi, Dominik Kowald, Denis Helic, Elisabeth Lex

**Affiliations:** 1 Know-Center GmbH, Research Center for Data-Driven Business & Big Data Analytics, Inffeldgasse 13/6, 8010 Graz, Austria; 2 Institute of Interactive Systems and Data Science, Graz University of Technology, Inffeldgasse 13/6, 8010 Graz, Austria

**Keywords:** Consensus dynamics, Online collaboration systems, Interaction networks, Similarity, Social status

## Abstract

**Background:**

In this paper, we study the process of opinion dynamics and consensus building in online collaboration systems, in which users interact with each other following their common interests and their social profiles. Specifically, we are interested in how users similarity and their social status in the community, as well as the interplay of those two factors, influence the process of consensus dynamics.

**Methods:**

For our study, we simulate the diffusion of opinions in collaboration systems using the well-known Naming Game model, which we extend by incorporating an interaction mechanism based on user similarity and user social status. We conduct our experiments on collaborative datasets extracted from the Web.

**Results:**

Our findings reveal that when users are guided by their similarity to other users, the process of consensus building in online collaboration systems is delayed. A suitable increase of influence of user social status on their actions can in turn facilitate this process.

**Conclusions:**

In summary, our results suggest that achieving an optimal consensus building process in collaboration systems requires an appropriate balance between those two factors.

## Background

In this work, we study opinion dynamics and consensus building in online collaboration systems. In collaboration systems such as online encyclopediae, question & answering (Q&A) sites or discussion forums, users engage in complex interactions with others to reach a common goal, such as to write an article or to answer a difficult question. Often, this is a long-lasting iterative process, in which users share their knowledge and opinions, discuss problems and solutions, write and edit joint articles, or vote on each others’ contributions. Ideally, this process converges to a shared common result. However, many times, consensus cannot be reached and a given topic or question remains unresolved within the community.

Understanding the factors, which govern a consensus building process in online collaboration systems, as well as mechanisms that may turn such a process into a success or failure is one of the pressing questions that our research community has already recognized. In many studies, researchers analyzed the underlying dynamics of opinion formation to identify key factors that contribute to consensus building (see, e.g., [1] for an overview). Such studies may be seen as a first step toward a more ambitious goal of developing tools that promote consensus building processes in online communities. For example, connecting otherwise non-interacting users by recommendations may lead to discussions resolving issues that hinder consensus.

To study consensus building processes, researchers frequently apply agent-based models. In an agent-based model, opinions of individual agents are represented as states and agents interact with each other following a set of predefined interaction rules. Through such interactions, agents alter their states until some criteria are met or the system reaches an equilibrium state. The interactions between agents give rise to a particular behavior of the whole population. The Naming Game model [2] is among the most prominent agent-based models for studying opinion dynamics and consensus building in groups of interacting agents. Often, such studies simulate opinion dynamics on synthetic networks, see for example [3–10].

In one of our previous works [11], we studied the influence of social status on consensus building in online collaboration systems. In that study, we assumed that the underlying network of previous interactions determines the constraints on the possible future interactions. In other words, only users who have already interacted with each other in the past were allowed to interact in the future. For example, user interactions on Reddit include users writing comments or voting on postings of other users. Such interactions allow us to extract user interaction networks from the system logs. In such networks, users are nodes and two users are connected by a link if they interacted in the past. However, in real-world online collaboration systems, there are certain user actions and interactions, which leave no or inconclusive traces in the system logs. For example, when users on Reddit simply read submissions but never leave comments or votes, in general we do not know which particular comments and postings these users actually have read. Moreover, many real-world datasets contain inaccuracies and are therefore inherently uncertain [12].

In this paper, we set out to study consensus building by adopting a model of interacting agents, whose future interactions are not restricted to the edges of the observed interaction network. Rather, we allow interactions between all pairs of users with varying preferences. In particular, we apply the Naming Game model and extend it to reflect (i) latent similarities between users and (ii) observed social status of users in real-world systems. Technically, with those two factors, we parametrize a probability distribution over pairs of users, which determines the likelihood of a future interaction between any two given users. This results in a flexible approach that enables us to explore and analyze various interesting and realistic configurations as opposed to restricting interactions to the edges of the observed network, which fixes the interaction probabilities to zero for previously non-interacting users.

To that end, we investigate consensus building within different society forms, which we characterize according to user similarity into *open*, *modular* and *closed* societies and according to social status into *egalitarian*, *ranked* and *stratified* societies. *Open* and *closed* societies represent two extreme cases based on the influence of user similarity: in an open society, any pairs of users can interact and exchange opinions with each other regardless of their similarity, whereas in a closed society only highly similar users interact with each other. Between these two society forms we define a *modular* society, in which probability of users’ interaction is proportional to their similarity. Similarly, *egalitarian* and *stratified* societies represent two extreme cases governed by configuring the influence of social status: in an egalitarian society, the influence of social status is neglected, indicating that users can interact and exchange opinions with each other regardless of their social status, whereas in a stratified society, opinions can flow only from users with a higher social status to those with a lower social status. Between these two extreme cases, we can model different situations (*ranked societies*) by tuning the influence of social status so that opinions are very likely to flow from individuals with a higher social status to those with a lower social status, but with small probability they can also flow into the other direction.

For our experiments, we extract 17 collaboration networks from the real-world systems Reddit and StackExchange. For each of these networks, we first determine user similarity and their social status. We determine user similarity by calculating their regular equivalence [13]. With regular equivalence, we capture global user similarities between non-interacting users as opposed to local similarity measures, which take into account only the immediate neighbors of a node. To determine social status of users, we use the built-in scoring schemes of Reddit and StackExchange. With these networks in place, we simulate opinion spreading among users to study how the process of consensus building is governed by configurable influences of user similarity, user social status and a complex interplay between those two factors.

The contributions of our work are twofold. First, we extend the Naming Game model with an interaction mechanism that is based on user similarities and their social status. With this extension, we conduct experiments on empirical collaboration networks and contribute in this way to the limited line of research on opinion dynamics in empirical networks. Second, our experimental results reveal interesting and non-trivial findings, namely, that user similarity and user social status are opposing forces with respect to consensus building. Whereas user social status may speed up the emergence of consensus, user similarities typically hinder that process. Thus, for an efficient consensus building the negative effect of similarity needs to be carefully compensated by the positive effect of social status.

## Related work

At present, we identify three main lines of research related to our work: (i) social impact theory, (ii) works that study the interplay between user similarity and social status and its impact on user behavior in online systems, and (iii) opinion dynamics in interaction networks.

### Social impact theory

In the field of social psychology, the social impact theory of Latané [14] attempts to explain how individuals are influenced by their social environments. According to it, the social impact felt by individuals can be explained in terms of social forces, to which they are exposed [15, 16]. Latané [14] compares these social forces to physical forces, such as electromagnetic forces or forces that govern the transmission of light, sound and gravity [15]. In this analogy, social forces felt by individuals are moderated by the strength, immediacy and number of other people present in their social environment. In relation to our work, the influence of users social status in our experiments refers to the strength of the impact of other people (e.g., their authority or power of persuasion), whereas the user similarity is analogous to the immediacy of the others (e.g., their closeness in space or time) [17]. Mathematically, the social impact felt by an individual, known also as a target, is a multiplicative function of the three features of a source person and is given in the following form: ${\text{Impact}} = f(S \cdot I \cdot N),$ where *Impact* is the social impact on the target person and *S*, *I*, and *N*, are the strength, immediacy and number of the source persons, respectively [14, 15]. The social impact function constitutes the theoretical basis for our agent-based model and its multiplicative effects.

Connecting the social impact theory with agent-based modeling has been also the aim of previous research [17], in which researchers applied computer simulations to examine the extent to which group-level phenomena are driven by individual-level processes. In synthetic datasets that represent sets of individuals, they studied the attitude change of individuals and group polarization with respect to binary opinion states. Similarly, in our work we apply agent-based modeling. However, we perform experiments on empirical datasets from online collaboration systems and consider more than two opinion states.

Recent work followed a theory-driven approach to conduct empirical analysis of Twitter data that supported the assumptions of the social impact theory [18]. In our work, however, we study the process of opinion dynamics in online collaboration systems, by applying a data-driven model as well as by simulating how opinions spread in those systems.

Cultural dynamics in society classes and their role on the adaption of fashion are the focus of the work of the sociologist Georg Simmel [19]. According to Simmel’s theory the latest fashion is defined by the higher society classes and the lower ones imitate and copy the fashion from them. As soon as this happens, higher classes move from the current fashion and adopt a new style to differentiate them from the masses. Similarly, in our analysis, we define higher and lower social status classes and analyze the opinion flow between them. The effect of lower status agents inflicting opinions to the higher ones, observed in our experiments, is comparable to the phenomenon of imitation, whereas the effect of limiting the communication from low-status agents to high-status agents reflects the phenomenon of differentiation.

The work presented in [20] applies an agent-based model to simulate the effects of Simmel’s theory by exploring its spatial dimension. While the authors use synthetic data and synthetic agent social statuses, we use empirical datasets from Reddit and StackExchange and apply the empirical reputation scores provided by both systems as a proxy for social status.

Research on how the position and social status of a node influence the network originates from network exchange theory [21–23]. Similarly, we study how the social status of a node in an interaction network affects the spread of opinion that leads to consensus building. Additionally, in our work we define classes of nodes based on the social status and determine how their interaction affects the process of consensus building.

### The influence of the interplay between user similarity and social status on user behavior in online systems

In our previous work [11], we studied the impact of social status on opinion dynamics and consensus building in online collaboration systems. In contrast, in the present work, we study how latent user similarity and the interplay between the user similarity and user social status impact the process of consensus building.

In [24] the authors present a framework for link prediction in evolving networks and show that popularity is just one dimension of attractiveness, in the context of link creation, and another important dimension is the similarity between users. In other words, user similarity and user popularity are two main forces that drive people to form links in various networks. In our work, we also study the effect of user similarity and user social status, but in relation to dynamical processes that take place in online collaboration systems.

User similarity in online social networks has also been studied in [25]. Here, the authors present a method for evaluating social networks according to network connections and profile attributes. In [26], the effect of similarity (in terms of user characteristics) and social status, as well as their interplay is studied on online evaluations carried out among users. They found that when two users are similar social status plays less of a role when users evaluate each other. Major difference to our work is that the authors calculate user similarity as cosine similarity between user action vectors. User actions are, for example, editing an article on Wikipedia, asking or answering a question on a Q&A site or rating a review on Epinions. In our work, we calculate user similarities by applying the regular equivalence that captures latent similarities even between non-interacting users and users who do not share common actions. Similar work to [26] is described in [27], with the difference that the authors consider only the relative social status between two users (i.e., their comparative levels of status in the group) when studying how users evaluate each other. The authors found that users with comparable status hesitate to give positive evaluations to each other.

### Opinion dynamics in interaction networks

Research on opinion dynamics in interaction networks builds upon insights from the field of statistical physics [1, 28]. In this field, social processes of interaction among individuals are modeled mathematically by representing how changes in the local and global state of an individual and a group take place. A well-known model following this approach, the Naming Game, has been introduced in the context of linguistics [2, 29] with the aim to demonstrate how autonomous agents can achieve a global agreement through pairwise communications without central coordination [30].

Recent research [9, 31] applies the mean field principle while using the Naming Game model for their experiments. For example, the work in [31] studies the impact of learning and the resistance toward learning (as two opposing factors) on consensus building among a population of agents. In [9], the authors consider the case of an arbitrary number of agent opinions and the presence of zealots in the Naming Game. They provide a methodology to numerically calculate critical points in two special cases: the case in which there exist zealots of only one type and the case in which there are an equal number of zealots for each opinion. Similarly to our approach, the work of Brigatti et al. [3] describes a variation of the Naming Game that incorporates the agent social status scores. In the beginning, social status is randomly distributed among the agents via a Gaussian distribution. Successful communication increases the agent social status and during each iteration, the agent with the higher social status acts as a teacher and the one with the lower status as a learner. In contrast to our work, the dynamic social status scores are synthetically created whereas we adopt empirical status scores.

## Methodology

We base our model on the Naming Game [2, 4, 32–34]. The Naming Game is an agent-based model, in which agents are represented as nodes in a network. Agents interact with each other by following a set of predefined rules, with the aim of giving a name to a single unknown object. Consensus is reached when all agents agree on a single name for the object.

Each agent possesses an inventory of names or words (i.e., opinions), which is initially empty. At each interaction step, two agents are randomly chosen to meet (i.e., to communicate), where one of them is designated the role of the speaker while the other one is the listener. If the speaker’s inventory is empty, a word is invented and it is communicated to the listener, or otherwise the speaker selects randomly a word from her inventory and communicates it to the listener. If the communicated word is unknown to the listener (i.e., it does not exist in the listener’s inventory), the listener adds this word to her inventory. Contrarily, if the communicated word is known to the listener, both speaker and listener agree on that word and drop all other words from their inventories.

In this work, we extend the Naming Game with an interaction mechanism that accounts for latent user similarities and social status. In [24], the authors have identified user similarity and user popularity as two main forces that drive people to form links in various networks. User similarity is a property of pairs of users whereas social status is a property of individual users. In general, in collaboration systems, users tend to connect with similar users, i.e., with those sharing similar interests, tastes or social backgrounds, as well as with users of a higher social status or a higher popularity [35].

### Regular equivalence

To calculate the user similarity, we apply similarity measures from graph theory and social network analysis. In these fields, there are two main types of similarity: (i) *structural similarity*, and (ii) * regular equivalence* [13]. In particular, two nodes in a network are structurally similar if they share many common neighbors. On the other hand, two nodes are regularly equivalent if they have common neighbors that are themselves similar even if they do not share the same neighbors. Thus, regular equivalence quantifies not only observable but also latent similarities.

With regular equivalence, the basic idea is to define a similarity score $\sigma _{ij}$ between nodes *i* and *j*, such that *i* and *j* are similar if *i* has a neighbor *k* that is similar to *j* [13]:

1 $$\begin{aligned} \sigma _{ij} = \alpha \sum _{k} A_{ik} \sigma _{kj} + \delta _{ij}, \end{aligned}$$

where $\alpha$ is a constant known as damping factor, $A_{ik}$ are elements of the adjacency matrix $\mathbf {A}$ (with $A_{ij}\ge 0$ if *i* and *j* are connected by an edge with a positive weight and $A_{ij}=0$ otherwise), $\sigma _{kj}$ is the similarity score between *k* and *j*, and $\delta _{ij}$ is the Kronecker delta function, which is 1 for $i=j$ and 0 otherwise. The damping factor $\alpha$ should satisfy $\alpha < 1/\kappa _{1}$ in order for similarity scores to converge, where $\kappa _{1}$ is the largest eigenvalue of the adjacency matrix. The recursive calculation of the regular equivalence counts paths of all lengths between pairs of nodes. It assigns high similarity values to nodes that either share many common neighbors or to nodes that are connected by many longer paths, or both. By choosing $\alpha$ closer to $1/\kappa _{1}$, we assign more weight to longer paths, whereas smaller $\alpha$ values prefer shorter paths. Since we want to capture as much of latent similarities as possible, we set $\alpha = 0.9/\kappa _{1}$.

**Fig. 1:**
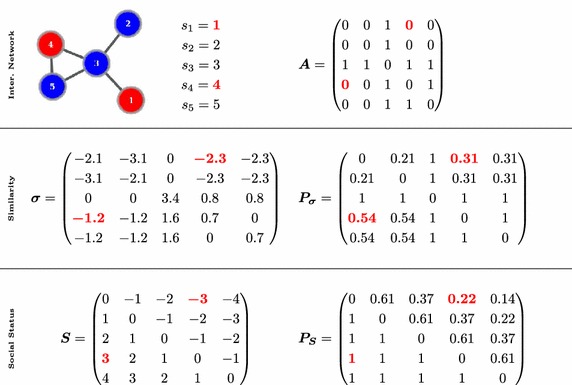
Probabilistic Meeting Rule—illustrative example. *Top row:* we depict an interaction network with five users, the social status of users ($s_1$ to $s_5$) and the adjacency matrix $\varvec{A}.$ All edge weights in $\varvec{A}$ are 1, indicating that the corresponding users interacted only once with each other in the past. If we restrict meetings to the edges of the interaction network, the meeting probabilities are symmetric and equal to the entries of $\varvec{A}.$ Thus, the users 1 and 4 cannot participate in a meeting since $p_{14}=p_{41}=0$ (elements marked in red in $\varvec{A}$). The average meeting probability $p_m$ corresponds to the network density and evaluates to 0.5. *Middle row:* we calculate the regular equivalence matrix $\varvec{\sigma }$ and normalize it with the degrees and the minimal neighbor similarity (normalization results in asymmetric similarities). We set closeness factor $\gamma =1/2$ (modular society) and calculate the matrix of meeting probabilities $\varvec{P_{\sigma }}$ (we set zeros on the diagonal since each meeting requires two users). The rows correspond to the meeting probabilities of a user acting as the speaker. We observe now non-zero probabilities between users who are not connected by an edge. For example, for users 1 and 4 (cf. red-marked elements in $\varvec{P_{\sigma }}$), the meeting probability is $p_{14}=0.31$ (user 1 acts as the speaker) and $p_{41}=0.54$ (user 4 acts as the speaker). In this setting, the average meeting probability is significantly higher than previously $p_m=0.69.$ *Bottom row:* the matrix $\varvec{S}$ keeps the (asymmetric) social status differences between users. Again, the rows correspond to users acting as the speaker in a meeting. We set stratification factor $\beta =1/2$ (ranked society) and calculate the matrix of the meeting probabilities $\varvec{P_S}.$ The social status mechanism results in non-zero probabilities between all pairs of users. For example, for users 1 and 4 (cf. red-marked elements in $\varvec{P_S}$), the meeting probability is $p_{14}=0.22$ (user 1 is the speaker) and $p_{41}=1$ (user 4 is the speaker). The average meeting probability for this configuration is $p_m=0.71.$ Finally, if similarity as well as social status rules apply, the final meeting probabilities are calculated by element-wise multiplication of $\varvec{P_\sigma }$ and $\varvec{P_S}$

The formula for similarity scores tends to give higher similarity to high-degree nodes due to their many neighbors [13]. A standard approach to remedy this situation is to normalize the scores by dividing them with the node degree.

Furthermore, we once more normalize the similarity values by subtracting for each user the minimum similarity of her direct neighbors. This lets us take into account the social adaptation of individual agents to the local norms induced by their neighbors [36]. As a result, we have positive similarity values only for the direct neighbors, as well as for all other users that are more similar than the direct neighbors (see Fig. 1 for an example of regular equivalence calculation).

### Probabilistic Meeting Rule

Algorithm 1 describes the procedure of our extension of the Naming Game. In particular, we modify the meeting rule between two agents and replace it with our Probabilistic Meeting Rule, which defines the probability of a meeting taking place:

2 $$\begin{aligned} p_{sl} = \underbrace{{\text{min}}\, (1, e^{\gamma \sigma _{sl}})}_{{{\text{similarity}}}} \cdot \underbrace{{\text{min}}\, (1, e^{\beta (s_s - s_l)})}_{{{\text{social status}}}}. \end{aligned}$$

Here, $\sigma _{sl}$ is the similarity score between speaker *s* and listener *l*, $s_s$ is the speaker’s social status, $s_l$ is the listener’s social status, $\gamma \ge 0$ is the *closeness factor* and $\beta \ge 0$ is the *stratification factor*. Note that those two factors are the controlling parameters in our model.

The Probabilistic Meeting Rule is a flexible rule that enables us to model various scenarios and society forms. The first term in the equation (${\text{min}}\, (1, e^{\gamma \sigma _{sl}})$) controls the degree of openness of a society. It evaluates to 1 for $\gamma =0$. We refer to this scenario as *open* society, in which any pair of users (mean field approach) can interact independently of how similar are they to each other. In other words, in an open society, the similarity between users does not play a role and everybody can interact with everyone else. Open society thus reflects the Solaria world introduced by Watts [37, 38].

By increasing $\gamma$, the influence of the user similarity becomes stronger indicating a so-called *modular* society (i.e., the first term in the Probabilistic Meeting Rule takes on a value between 0 and 1). In this scenario, highly similar users interact with each other with a high probability, whereas less similar users still interact with each other but with a smaller probability than highly similar users. By further increasing the closeness factor we arrive at a *closed* society, in which users interact only with other highly similar users and the interaction probability between less similar users evaluates to a value close to 0. This scenario is analogous to the Watts’ caveman world, in which users who live in “caves” (i.e., closed communities) interact with each other but they never or rarely interact with users from other “caves” [37, 38].

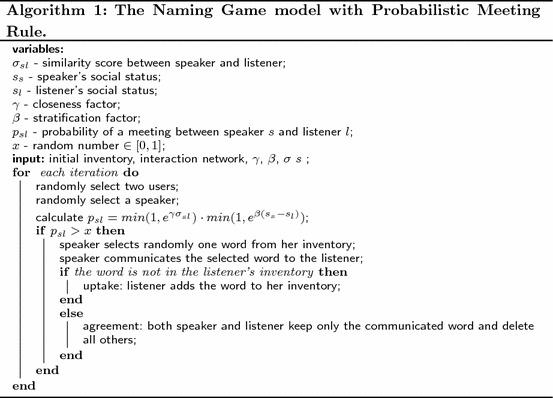



Similarly, with the stratification factor, we can configure the level of influence of the users social status on the probabilities of their interactions. In particular, if the speaker’s social status is higher than the listener’s social status, the second term (${\text{min}}\, (1, e^{\beta (s_s - s_l)})$) in Eq. 2 takes the value of 1. This means that a meeting between a speaker with a social status higher than the listener’s always takes place. When the listener has a higher social status than the speaker, several scenarios are possible, depending on the value of the stratification factor. For example, for $\beta =0$, the second term evaluates to 1 and we call this scenario *egalitarian* society. In an egalitarian society, everyone can talk to everyone else independently of their social status. If we increase the stratification factor, the second term starts to decay and in general, takes a value between 0 and 1. We refer to this situation as a *ranked* society, in which opinions always flow from individuals with a higher social status to those with a lower social status. Flow into the other direction is also possible, however only with small probability. By further increasing $\beta$, we reach a situation where the second term always evaluates to a value close to 0 if the speaker’s social status is smaller than the listener’s social status. In other words, we have reached what we call a *stratified* society where meetings take place only if the speaker’s social status is higher than the listener’s social status but never in the opposite case. Thus, with varying configurations of both terms, we can explore nine different combinations of the above-mentioned scenarios.

In Fig. 1, we show an illustrative example for the calculation of the meeting probabilities for a modular, ranked society. In general, we observe two effects of our approach: (i) the meeting probabilities increase as compared to a model which restricts interaction to the edges of the interaction network, and (ii) the meeting probabilities are asymmetric.

## Datasets and experiments

In our experiments, we use 17 empirical datasets from Reddit and StackExchange by selecting them randomly to ensure a broad coverage of different topics.

### Extracting interaction networks

In Reddit, registered users post new submissions (typically links or texts), comment and discuss existing submissions, or create new communities (so-called subreddits), which revolve around a specific topic. For our experiments, we parsed the dumps of 16 different subreddits from the year 2014, which belong to four main categories^1^: *Movies* (*Documentaries*, *True film*, *Movie details* and *Harry Potter*), *Politics* (*Political discussion*, *Political humor*, *Neutral politics* and *World politics*), *Programming* (*Julia*, *Python*, *Ruby* and *Compsci*) and *Sports* (*Skiing*, *Tennis*, *Badminton* and *Volleyball*). To construct the Reddit interaction network, we extract the users’ contributions from the submission^2^ and from the comment^3^ dumps. We then create an interaction network, in which users are represented as nodes and two users are connected by an edge if one user commented on the submission of another one, or if they both commented on the same submission of a third user. For each edge, we set a weight, which corresponds to the number of interactions between two given users.

StackExchange^4^ is a Q & A site, where users collaboratively solve problems through asking and answering questions in posts. Similarly to the Reddit networks, we construct the StackExchange interaction networks to represent co-posting activities. Specifically, two nodes (i.e., users) are connected via a weighted edge if the users contributed to the same question. Correspondingly, the edge weight encodes the number of common contributions. We use the following StackExchange editions covering different topics for our experiments: *English, Cooking, Academia, Movies, Politics, Music, German, Japanese, History, Chinese, Spanish, French, Sports*.^5^

Finally, in all networks, we extract the largest connected component and perform all experiments on that component. We give the basic statistics of our empirical datasets such as the number of nodes (*n*) and edges (*m*), as well as average node degree (*d*), average social status (*s*), average edge weight (*e*) and density ($\rho$) in Table 1. The network density $\rho$ calculated as $2m/(n(n-1))$ is defined as the fraction of all possible edges that are present in a network. In the context of our model, density can be interpreted as an average meeting probability if meetings are restricted only to the edges of the network. In other words, the probability that a randomly selected pair of users participates in a joint meeting equals, on average, to the network

**Table 1:** Dataset characteristics

Dataset	Type	*n*	*m*	*d*	*s*	*e*	$\rho$
Reddit	Movies	38,006	138,907	7.3	6	1.1	0.00019
	Politics	25,946	92,285	7.1	8.2	1.2	0.00027
	Programming	23,074	70,232	6.1	5.4	1.1	0.0003
	Sports	18,441	97,073	10.5	10.2	1.2	0.00057
StackExchange	English	30,656	192,983	12.5	199	1.7	0.0004
	Cooking	9637	40,437	8.4	175	1.6	0.0009
	Academia	5098	26,805	10.5	312	1.7	0.002
	Movies	4425	13,952	6.3	194	1.8	0.0014
	Politics	4349	21,428	9.8	229	2	0.002
	Music	3699	15,750	8.5	213	1.7	0.0023
	German	2316	12,825	11	285	2.2	0.0048
	Japanese	2069	11,155	10.7	328	2.5	0.0052
	History	2054	11,048	10.7	271	2.2	0.0052
	Chinese	1985	8556	8.6	160	1.8	0.0043
	Spanish	1584	6908	8.7	196	1.9	0.0055
	French	1478	6668	9	298	2.1	0.0061
	Sports	1276	3513	5.5	178	1.8	0.0043

This table shows the number of nodes (*n*), number of edges (*m*), average node degree (*d*), average social status (*s*), average edge weight (*e*) and density ($\rho$) of our networks

density. In practice, the majority of social and other networks such as interaction networks are extremely sparse networks with densities that lay way beyond 1%. Thus, our empirical interaction networks indeed constitute a very rigid constraint on any possible interactions.

###  Determining social status

To determine the social status scores for users, we exploit the built-in user rewarding system of Reddit and StackExchange. In Reddit, users can accumulate so-called “karma” scores that rise if their posts receive good ratings from other users. Thus, karma scores represent the reflection of the user “vibes” in the community and we apply it as a proxy for social status. Since karma scores are not included in the publicly available Reddit dumps, we crawled those scores using the public API^6^ and the python-based PRAW API wrapper.^7^ On the other hand, in StackExchange users are rewarded by the community with reputation scores for providing not only valuable answers but also valuable questions. As shown in [39], the scores given by this user-rewarding system highly correlate with the quality of the user content and thus, we assume that a high-reputation user contributes with a high-quality content to the community. Reputation scores are provided in the dataset dumps and we use them as a proxy for social status in StachExchange systems. This setup allows us to investigate the effect of social status from two view points: in Reddit, the social status is a reflection of how other persons experience a given user in the society (i.e., charisma) and in StackExchange, social status is more related to a position that users earn in a society based on the quality of their work (i.e., reputation).

### Experiments

Our experiments consist of six steps. First, for each interaction network, we construct a weighted adjacency matrix $\mathbf {A}$ by setting $A_{ij}$ to the edge weight between users *i* and *j*, if they are connected or to 0 otherwise. Second, we compute the matrix of similarity scores using the methodology described in “Methodology” section.

Third, we compute the closeness factor $\gamma$ and the stratification factor $\beta$ by estimating the expected meeting probability in our networks using a standard Monte Carlo method [11]. This enables us to control the communication intensity between users. For the closeness factor, we determine two parameter values to depict modular and close societies by controlling the percentages of successful meetings induced by the first similarity factor in our multiplicative Probabilistic Meeting Rule: (i) for the modular society, we determine $\gamma$ such that approximately 75% of all possible meetings (up to the statistical fluctuations) take place, (ii) for the closed society, we determine $\gamma$ for which approximately half of all meetings are successful on average. In addition, for the open society, in which all meetings take place, we set $\gamma =0.$

Average meeting probability of 50% is 2 orders of magnitude higher than the average network density of our empirical interaction networks (0.27%) (cf. Table 1). Thus, even though our model biases the user interactions toward more similar users, it is substantially less restrictive than an alternative model solely based on the interaction network. Another (simpler) alternative to avoid the restrictions of the interaction networks would be to, for example, allow for each second interaction to take place between arbitrary pairs of (non-adjacent) users. Nevertheless, this approach would miss the possibility to induce similarity or social status biases.

Similarly to the closeness factor, we also estimate two values for the stratification factor $\beta$ that correspond to the ranked and stratified society forms. Here, we control the opinion flow from low to high social status users and set $\beta$ such that on average, 50% of meetings take place (ranked society) and so that none of the meetings takes place (stratified society) (again we only control the second social status factor in the multiplicative meeting rule). In addition, by setting $\beta =0$ we achieve the egalitarian society, in which all meetings take place. Note that we define high social status users as users with a social status above the 90th percentile, whereas low social status users have a social status below the 90th percentile.

Fourth, we initialize agents’ inventories by randomly selecting three words from a set of unique words for each agent. Fifth, we create a set of meetings, i.e., randomly selected pairs of users. From this set, we generate meeting sequences by picking meetings at random without repetition for each possible combination of closeness factor and stratification factor. This ensures that the random factor due to the meeting sequence remains insignificant for various values of $\gamma$ and $\beta.$ We determine the length of the meeting sequence (*c*) (i.e., maximum number of user interactions) based on the number of users in a given dataset. The length of the meeting sequence *c* is 2 orders of magnitude higher than the number of users *n*. For each configuration, we simulate the meetings 100 times and report the averaged simulation results.

Finally, we store the state of the agents’ network for each *c*/100 interaction of our 100 runs as well as for all values of closeness factor and stratification factor. This enables us to investigate the distinct number of overall opinions adopted by each agent during the interactions. Additionally, we can derive the percentages of agents that have reached consensus on a particular opinion.

#### Source code

To ensure the reproducibility of our experiments, we provide our simulation framework as an open-source project. The source code can be downloaded from our Git repository.^8^

## Results and discussion

###  The influence of user similarity and social status on consensus dynamics

**Fig. 2:**
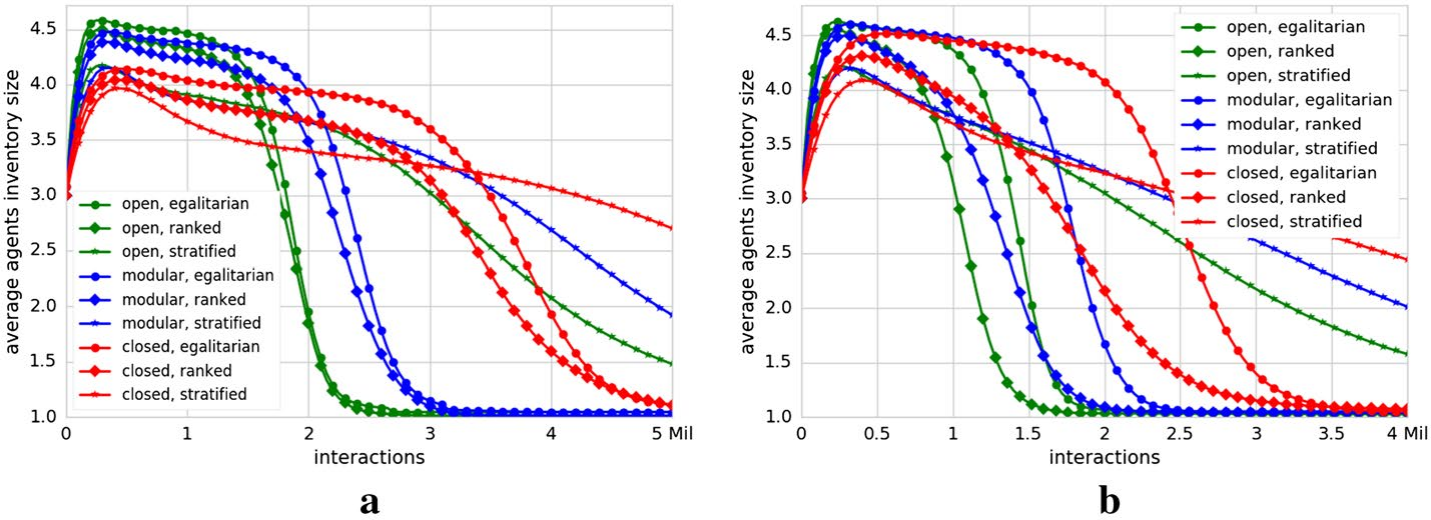
The influence of user similarity and social status on consensus dynamics. The plots show the mean size (100 runs) of the agents’ inventories (*y*-axes) in relation to the number of interactions (*x*-axes) for Reddit Movies (**a**) and StackExchange English (**b**) datasets. Each line represents results for one particular $\gamma$ and $\beta$. The line colors represent three values of $\gamma$ and line markers three different values of $\beta.$ We notice that in (*modular, egalitarian*) and (*closed, egalitarian*) societies (marked with blue and red lines with circle markers), opinion convergence rates are slower than in (*open, egalitarian*) society marked with green and circle markers. This indicates that as soon as user similarity plays a role, consensus building is delayed. However, in (*modular, ranked*) and (*closed, ranked*) societies (blue and red lines with diamond markers) we observe faster consensus building. This means that by increasing the effect of social status, we are able to partially compensate the negative effect of similarity. By further increasing the impact of the social status through the stratification factor, the positive effect of social status dissolves. This is visible in the green, blue and red lines with star markers corresponding to (*open, stratified*), (*modular, stratified*) and (*closed, stratified*) societies. Thus, for a faster consensus building, a careful balancing between the influence of similarity and social status is needed.** a** Reddit Movies,** b** StackExchange English

We show our simulation results in Fig. 2. The plots in Fig. 2a, b depict the evolution of the agents’ inventory mean size (over 100 runs) as a function of the simulation progress for the Reddit Movies and StackExchange English datasets, respectively. All other empirical datasets exhibit comparable results, so we omit them from Fig. 2; but we provide them in Appendix in Fig. 5. Each line in the plots corresponds to the results obtained using one particular closeness factor and stratification factor. Line colors depict different values of closeness factor, whereas line markers illustrate values of stratification factor.

Due to our Probabilistic Meeting Rule, whenever we set one of the factors to 0, we can study the impact of the other factor on the process of consensus building. Thus, by analyzing society forms with $\beta =0$ (*egalitarian*) and varying closeness factor, we can investigate the effect of user similarity on the consensus building process. Our results reveal that in *(modular, egalitarian)* and *(closed, egalitarian)* societies (cf. blue and red lines with circle markers in Fig. 2) consensus is slowed down as compared to *(open, egalitarian)*, which represents a society where all meetings take

place. Thus, as soon as user similarity starts to exhibit influence on the meeting probabilities the consensus building process is delayed. Although the average meeting probability in *modular* society forms is still very high, even this slight preference toward meeting with more similar users is able to slow down the spread of opinions.

On the other hand, a weak increase in the influence of the user social status is beneficial for the consensus. In *(modular, ranked)* and *(closed, ranked)* societies (cf. blue and red lines with diamond markers in Fig. 2), we observe faster consensus building. Thus, by increasing the effect of social status, we can compensate the initial negative effect of similarity.

Nevertheless, the positive effect of social status diminishes quickly. In *(modular, stratified)* and *(closed, stratified)* societies (cf. blue and red lines with star markers in Fig. 2), the convergence rate again slows down. Thus, an initially positive effect of social status in ranked society forms undergoes a phase transition toward a negative effect in stratified societies.

#### Findings

Our simulation results indicate that user similarity and social status exhibit opposing forces with respect to consensus building in online collaborative systems. While an increase in the influence of user similarity has a negative effect, the social status exhibits both the phase of a positive effect as well as the phase of a negative effect. Consequently, an optimal configuration for a faster consensus requires a careful balance between those two factors.

###  Coarse analysis

We consider the average inventory size of agents equalling 1 as a first criterion for reached consensus among agents (cf. Fig. 2). Further, we aim to determine the distinct number of opinions present in the agents network and the consensus strength during the interactions. We define the consensus strength as percentages of agents having one single opinion in their inventories over the course of simulations. The consensus strength reaches its maximum when all agents unanimously agree on one particular opinion.

**Fig. 3:**
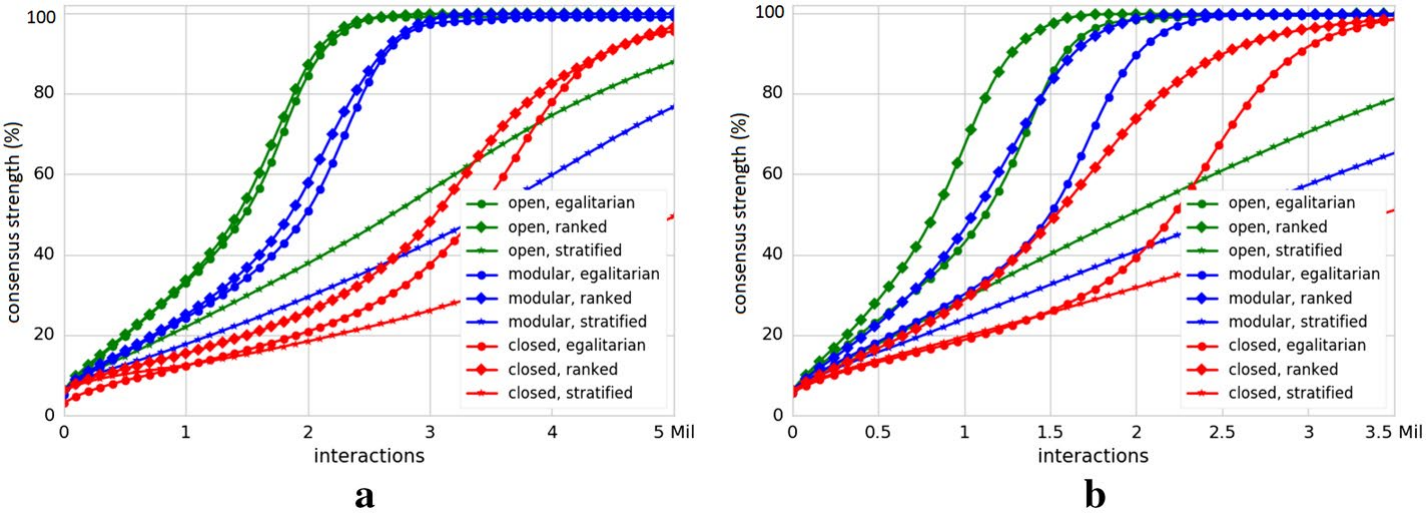
Coarse analysis. Percentages of consensus strength (averaged over 100 runs) reached among agents on one particular opinion (*y*-axes) are shown as a function of the number of interactions (*x*-axes) for different values of $\gamma$ and $\beta.$ The line colors illustrate three different values of $\gamma$ and line markers three values of $\beta.$ The plot in** a** illustrates Reddit Movies results, whereas the plot in** b** presents the results of StackExchange English dataset. Each line represents results for one particular configuration of $\gamma$ and $\beta$. We consider that the consensus strength reaches its maximum when all agents unanimously agree on one particular opinion. With each interaction, agents exchange opinions and the consensus strength increases, but with different growth rates for different configurations of $\gamma$ and $\beta$. The growth rates of the consensus strength lines determine how quickly agents reach consensus. A saturation of consensus strength lines is visible for almost all society forms except for *(open, stratified)*, *(modular, stratified)* and *(closed, stratified)* (cf. green, blue and red lines with star markers). The growth rates are faster for *(open, ranked)*, *(modular, ranked)* and *(closed, ranked)* (cf. green, blue and red lines with diamond markers) compared to *(open, egalitarian)*, *(modular, egalitarian)* and *(closed, egalitarian)* societies (cf. green, blue and red lines with circle markers). These results complement our previous findings presented in Fig. 2 and reveal that the appropriate balance between user similarity and social status enables faster consensus strength growth rates in online collaborative systems.** a** Reddit Movies,** b** StackExchange English

Figure 3 shows consensus strength (averaged over 100 runs) over the number of interactions for the Reddit Movies (Fig. 3a) and StackExchange English (Fig. 3b) datasets. Analogous to Fig. 2, each line represents results for one particular $\gamma$ and $\beta.$ The line colors represent three values of $\gamma$ and line markers three different values of $\beta.$

For almost all societies except for *(open, stratified)*, *(modular, stratified)* and *(closed, stratified)* (cf. green, blue and red lines with star markers in Fig. 3), there is a saturation of the consensus strength visible in the plots. The growth curves resemble logistic growth curves with the phases of quick initial growth and a saturation phase as the process reaches its equilibrium. The growth rates of the consensus strength lines determine how quickly agents reach consensus. The growth rates are faster for *(open, ranked)*, *(modular, ranked)* and *(closed, ranked)* (cf. green, blue and red lines with diamond markers) compared to *(open, egalitarian)*, *(modular, egalitarian)* and *(closed, egalitarian)* societies (cf. green, blue and red lines with circle markers). These results complement our findings presented in the previous

subsection, namely, with the increase of the influence of user similarity on the meeting probabilities, consensus building among agents is delayed. This negative effect is compensated to some extent with the increase of the influence of social status (*ranked societies*). A further increase of the influence of social status yet hinders consensus building, which means that no saturation state can be observed in case of *stratified* societies (at least not in the number of interactions that we simulate).

#### Findings

Our coarse analysis reveals that the optimal balance between user similarity and social status enables faster growth rates toward consensus building in our datasets.

### Communication intensity between social classes

Now we are interested in identifying causes of these observed effects. For this, we investigate the communication intensity (i.e., the number of successful meetings) across user social classes that we introduced earlier, namely high social status class with users above the 90th percentile and low social status class with all other users.

In our previous study [11], we found that the direction of opinion flow impacts how fast opinions converge. Specifically, the flow from low social status to high social status users, as well as from low social status users to low social status users, is crucial. As described in [40], high social status users are typically able to impose their opinions to other users in a system. Thus, whenever the opinions of these high social status users frequently change the system as a whole experiences oscillatory behavior and cannot reach consensus. Due to the heterogeneous distributions of user social status in many systems, the number of low social status users is substantially higher than the number of high social status users. Therefore, whenever the communication intensity in the direction from low social status users to high social status users is high, low social status users are able to cause oscillations in the opinions of high social status users and the consensus building process is delayed.

On the other hand, it is important that communication direction from low social status users to other low social status users remains unhindered. Due to the high number of low social status users, they have to be able to intensely communicate among themselves to spread opinions. Low social status users cannot rely on a small number of high social status users to reach many low social status users and distribute opinions. In other words, the process of consensus building among low social status users is a two-phase process. First, high social status users impose their opinions onto a small fraction of low social status users, and second, those opinions are subsequently spread among low social status users themselves.

These mechanisms can potentially explain the results of our experiments. For example, due to their numerous previous interactions high social status users are on average more similar to other users than low social status users. Therefore, whenever user similarity is the driving force behind meetings taking place we expect users with high social status to participate in a large number of meetings.

On the other hand, the number of low social status users is high and the second meeting participant is very likely a low social status user. Thus, our expectation is that we will observe many meetings with one high social status and one low social status user. This increases the communication intensity between low and high social status users and this leads to increased opinion fluctuations for high social status users. This in turn can slow down the consensus building process.

To further investigate this hypothesis, we analyze the percentages of users’ interactions that turn into successful meetings after applying our Probabilistic Meeting Rule. Specifically, we analyze two important communication directions and their intensities: (i) low-to-high and (ii) low-to-low, where the first term refers to the speaker and the second to the listener.

**Fig. 4:**
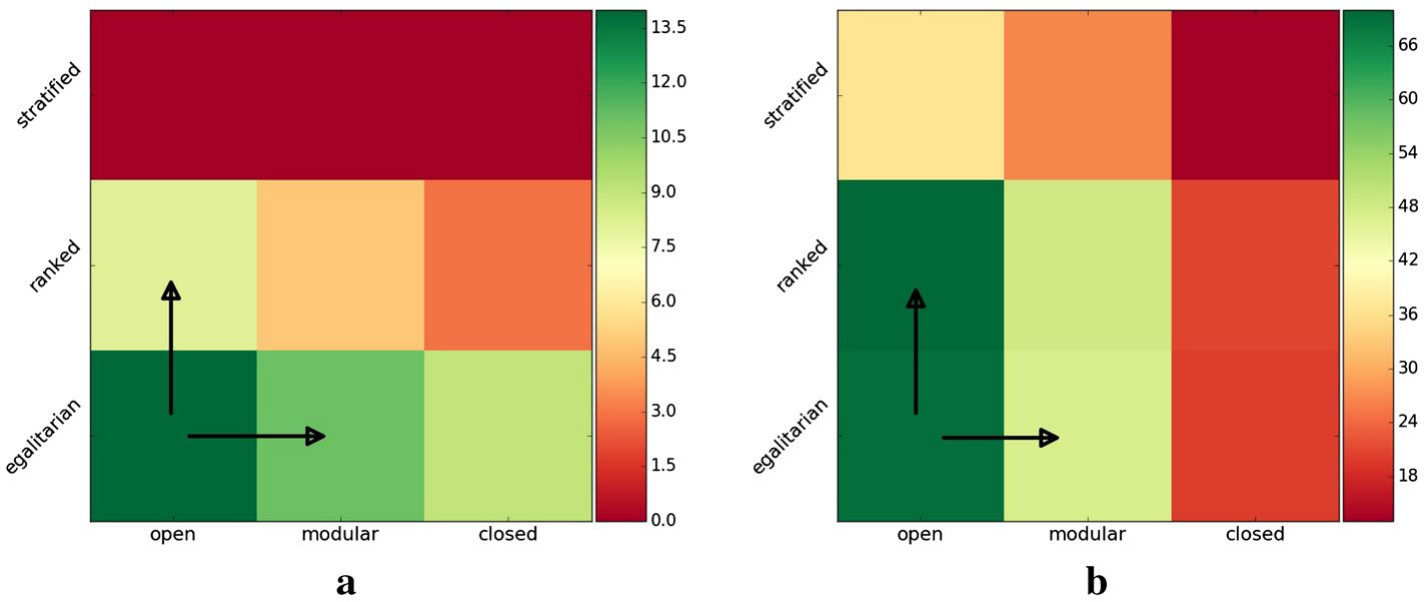
Heatmaps of the communication intensity in (**a**) low-to-high and (**b**) low-to-low social status classes of users for the StackExchange English dataset. The columns represent three society forms based on similarity: open, modular and closed, whereas rows show three social status society forms: egalitarian, ranked and stratified. The colors depict the intensity of the communication between users (i.e., percentages of the successful meetings taking place). In the plot in **a**, we notice that the communication intensity from low- to high-status users is decreased by increasing either the influence of user similarity (switch from open to modular society) or the social status (switch from egalitarian to ranked society). In **b**, we see that by switching from an open to a modular society the communication intensity from low- to low-status users is decreased. But for optimal consensus building, the communication in this class of users should not be disturbed. When we switch from an egalitarian to a ranked society, the intensity of the communication between users in the low-to-low class remains unchanged. This is one of the factors that in the ranked societies we observe fast opinion convergence rates. To summarize, through the increase in similarity the communication channel from low-status users to other low-status users is being closed and this slows down the consensus building process.** a** Low-to-High,** b** Low-to-Low

**Fig. 5:**
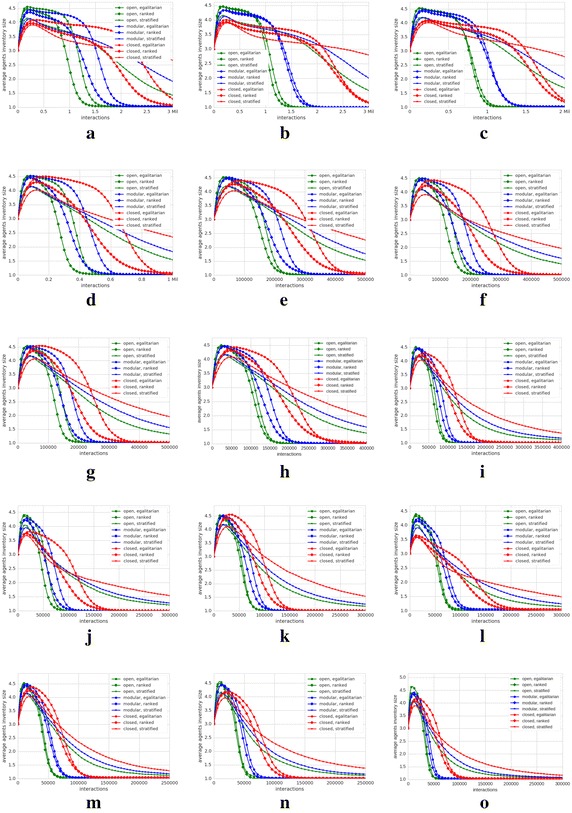
The influence of user similarity and social status on consensus dynamics. The plots show the mean size (100 runs) of the agents’ inventories (*y*-axes) in relation to the number of interactions (*x*-axes) for all datasets from Table 1 not included in Fig. 2. The simulation results are similar to the ones presented in Fig. 2.** a** Reddit Politics,** b** Reddit Programming,** c** Reddit Sports,** d** StackExchange Cooking,** e** StackExchange Academia,** f** StackExchange Movies,** g** StackExchange Politics,** h** StackExchange Music,** i** StackExchange German,** j** StackExchange Japanese,** k** StackExchange History,** l** StackExchange Chinese,** m** StackExchange Spanish,** n** Stackexchange French,** o** StackExchange Sports

**Fig. 7:**
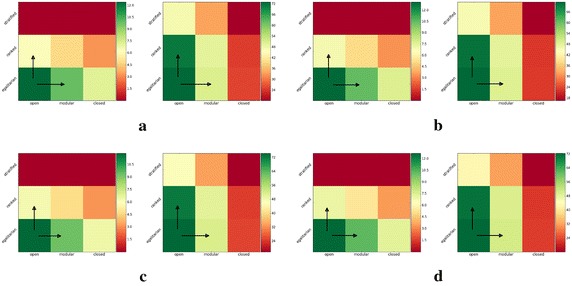
Communication intensity between social classes. Heatmaps of the communication intensity in low-to-high (left) and low-to-low (right) social status classes of users for all Reddit datasets from Table 1 not included in Fig. 4. The results are very similar to those presented in Fig. 4. ** a** Reddit Movies,** b** Reddit Politics,** c** Reddit Programming,** d** Reddit Sports

**Fig. 8:**
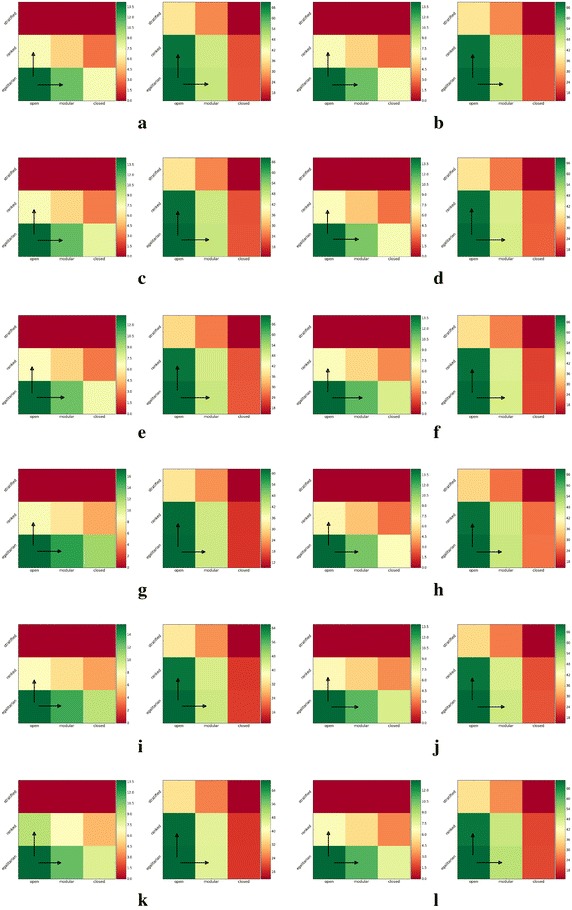
Communication intensity between social classes. Heatmaps of the communication intensity in low-to-high (left) and low-to-low (right) social status classes of users for StackExchange datasets from Table 1 not included in Fig. 4. The results are very similar to those presented in Fig. 4. ** a** StackExchange Cooking,** b** StackExchange Academia,** c** StackExchange Movies,** d** StackExchange Politics,** e** StackExchange Music,** f** StackExchange German,** g** StackExchange Japanese,** h** StackExchange History,** i** StackExchange Chinese,** j** StackExchange Spanish,** k** Stackexchange French,** l** StackExchange Sports

In Fig. 4, we show a heatmap with communication intensities between social classes for all nine combinations of society forms for the StackExchange English dataset. Again, here we only present the results for this dataset, since in all other datasets we obtain comparable results; we provide them in Appendix in Figs. 7 and 8. The heatmap in Fig. 4a depicts the percentages of successful meetings in the low-to-high class of users, whereas the heatmap in Fig. 4b depicts the percentages of successful meetings taking place in the low-to-low class. Columns of the heatmaps show the society forms based on similarity (i.e., open, modular and closed) and rows show the social status society forms (i.e., egalitarian, ranked and stratified).

The communication intensity from low to high social status users (cf. Fig. 4a) is decreased when either the influence of user similarity (switch from open to modular society) or social status (switch from egalitarian to ranked society) is increased. In the ranked society, we observe a slightly higher reduction in the opinion flow from low to high social status users than in the modular society. Thus, even though high social status users are on average more similar to other users, increase in the influence of similarity reduces the opinion flow from low social status to high social status users. Since this is a desired behavior there seems to be some other mechanism causing the delay in the opinions convergence.

Therefore, we turn our attention now on the low-to-low communication direction. By switching from an open to a modular society, we observe a decreasing opinion flow from low to low social status users (cf. Fig. 4b). However, for optimal consensus building, the communication in this class of users should not be disturbed. On the other hand, when we switch from an egalitarian to a ranked society, the intensity of the communication between users in the low-to-low class remains unchanged and we observe fast convergence rates. Thus, through the increase in similarity the communication channel from low social status users to other low social status users is being closed and this slows down the consensus building process. Similar behavior can be also observed for the social status when we switch from ranked to stratified society form. Thus, a balanced influence of social status improves convergence rates, whereas even a low influence of similarity hinders the process.

#### Findings

Our analysis indicates that the increased influence of similarity reduces the communication intensity between both low social status users and high social status users, as well as between low social status users and other low social status users. While the former has a positive effect on the spreading of opinions the latter hinders that process and causes the delay in consensus. Meetings governed by similarity are locally contained to smaller groups of users and the communication between different users groups is less intensive.

## Conclusion and future work

In this paper, we studied the process of opinion dynamics and consensus building in online collaboration systems. Specifically, we adopted a model of interacting agents, in which we allow interactions between all pairs of users with varying preferences beyond the observed interaction network. To that end, we presented an extension to the Naming Game model, i.e., the Probabilistic Meeting Rule that reflects (i) latent similarities between users and (ii) observed social status of users in real-world systems. We conducted our experiments on 17 empirical datasets from Reddit and StackExchange.

Our experimental results revealed that user similarity and social status exhibit opposing forces with respect to consensus building in online collaborative systems. Our main finding indicates that while an increase in the influence of user similarity has a negative effect, social status exhibits both the phase of a positive effect as well as the phase of a negative effect. Consequently, for a faster consensus, a careful balance between those two factors is required.

To explain our results, we further investigated the communication intensity (i.e., the number of successful meetings) between the social classes we defined. Our findings showed that the increased influence of similarity reduces the communication intensity between both low-status users and high-status users, as well as between low-status users and other low-status users. While the former has a positive effect on the spreading of opinions the latter hinders that process and causes the delay in consensus.

### Limitations

In our opinion, our work has the following limitations. First, we neglected any dynamic changes of user similarity and social status and the networks as such. In reality, social networks constantly change as users may leave the system while others join. We could gain more realistic insights by comparing results of dataset snapshots between different points in time. Second, we used a simplification for opinions of users exchanged in online collaboration networks by presenting them as a set of numbers. An alternative would be to use the real content exchanged among users.

### Future work

For future work, we plan to use our insights to design personalized user recommendation algorithms. Thus, by identifying the factors that lead to barriers and conflicts in collaborations, we plan to design meaningful interventions by suggesting possible collaborators with the goal to create network structures, in which consensus building is supported (i.e., recommending experts or high social status users as possible collaborators with the goal to speed up the process of consensus building). We also plan to verify our findings in other types of empirical networks, for example, gathered from the collaborative editing system Wikipedia, where we will investigate the dynamics of the editing process.

## Figures and Tables

**Fig. 6:**
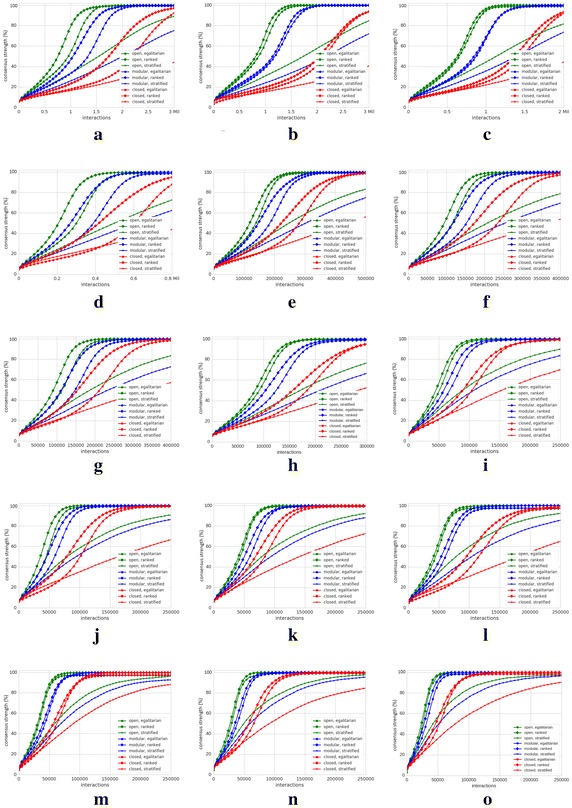
Coarse analysis. Percentages of consensus strength (averaged over 100 runs) reached among agents on one particular opinion (*y*-axes) are shown as a function of the number of interactions (*x*-axes) for different values of $\gamma$ and $\beta$ for all datasets from Table 1 not included in Fig. 3. Coarse analysis results are similar to the ones presented in Fig. 3. ** a** Reddit Politics,** b** Reddit Programming,** c** Reddit Sports,** d** StackExchange Cooking,** e** StackExchange Academia,** f** StackExchange Movies,** g** StackExchange Politics,** h** StackExchange Music,** i** StackExchange German,** j** StackExchange Japanese,** k** StackExchange History,** l** StackExchange Chinese,** m** StackExchange Spanish,** n** Stackexchange French,** o** StackExchange Sports

## Appendix

See Figs. 5, 6, 7, 8.

## Footnotes

1 https://www.reddit.com/r/ListOfSubreddits/wiki/listofsubreddits/.

2 https://www.reddit.com/r/datasets/comments/3mg812/full_reddit_submission_corpus_now_available_2006/.

3 https://www.reddit.com/r/datasets/comments/3bxlg7/i_have_every_publicly_available_reddit_comment/.

4 https://stackexchange.com/.

5 https://archive.org/details/stackexchange.

6 https://www.reddit.com/dev/api/.

7 https://praw.readthedocs.io/en/stable/.

8 https://git.know-center.tugraz.at/summary/?r=SocialNetworkAnalysis.git.
